# Oligometastatic Disease Management: Finding the Sweet Spot

**DOI:** 10.3389/fonc.2020.617793

**Published:** 2020-12-22

**Authors:** Petr Szturz, Daan Nevens, Jan B. Vermorken

**Affiliations:** ^1^ Medical Oncology, Department of Oncology, Lausanne University Hospital (CHUV), Lausanne, Switzerland; ^2^ Department of Radiation Oncology, IridiumNetwork, Wilrijk (Antwerp), Belgium; ^3^ Faculty of Medicine and Health Sciences, University of Antwerp, Antwerp, Belgium; ^4^ Department of Medical Oncology, Antwerp University Hospital, Edegem, Belgium

**Keywords:** head and neck cancer, oligometastatic, metastasectomy, surgery, stereotactic ablative body radiotherapy, immunotherapy, surveillance, cure

## Abstract

Hematogenous dissemination represents a common manifestation of squamous cell carcinoma of the head and neck, and the recommended therapeutic options usually consist of systemically administered drugs with palliative intent. However, mounting evidence suggests that patients with few and slowly progressive distant lesions of small size may benefit from various local ablation techniques, which have already been established as standard-of-care modalities for example in colorectal and renal cell carcinomas and in sarcomas. In principle, serving as radical approaches to eradicate cancer, these interventions can be curative. Their impact on local control and overall survival has been shown in numerous retrospective and prospective studies. The term oligometastatic refers to the number of distant lesions which should generally not surpass five in total, ideally in one organ. Currently, surgical resection remains the method of choice supported by the majority of published data. More recently, stereotactic (ablative) body radiotherapy (SABR/SBRT) has emerged as a viable alternative. In cases technically amenable to such local interventions, several other clinical variables need to be taken into account also, including patient-related factors (general health status, patient preferences, socioeconomic background) and disease-related factors (primary tumor site, growth kinetics, synchronous or metachronous metastases). In head and neck cancer, patients presenting with late development of slowly progressive oligometastatic lesions in the lungs secondary to human papillomavirus (HPV)-positive oropharyngeal cancer are the ideal candidates for metastasectomy or other local therapies. However, literature data are still limited to say whether there are other subgroups benefiting from this approach. One of the plausible explanations is that radiological follow-up after primary curative therapy is usually not recommended because its impact on survival has not been unequivocal, which is also due to the rarity of oligometastatic manifestations in this disease. At the same time, aggressive treatment of synchronous metastases early in the disease course should be weighed against the risk of futile interventions in a disease with already multimetastatic microscopic dissemination. Therefore, attentive treatment sequencing, meticulous appraisal of cancer extension, refinement of post-treatment surveillance, and understanding of tumor biology and kinetics are crucial in the management of oligometastases.

## Introduction

Recent therapeutic achievements in head and neck cancer managed to reduce the risk of death from recurrences and metastatic dissemination or at least contributed to delaying disease progression and quality of life deterioration. Apart from new systemic modalities leveraging the immune cells to combat cancer, increasing attention has been drawn towards local ablative approaches, which either as complementary or stand-alone therapies demonstrated encouraging activity against distant lesions. In particular, cases with few slowly growing metastases seem to constitute the ideal candidates (1). These patients present with different forms of oligometastatic disease. Besides a de-novo diagnosis, it may develop in the context of a controlled primary tumor (oligorecurrence) or otherwise controlled polymetastatic disease (oligoprogression). Herein, we will discuss the current state of the art in management of oligometastatic head and neck cancer in order to assist physicians in finding the optimal spot in the disease course where such treatment brings the maximum benefit to patients. However, before doing that, we will briefly review some important facts about metastatic outgrowth defining the patients at risk, addressing different diagnostic methods, and introducing available treatment options.

## Distant Metastases: Who, How, and What

*Who is at risk of developing distant lesions?* Compared with other malignancies, the proportion of head and neck cancer patients presenting with hematogenous dissemination is generally smaller and varies from 3%–17% at presentation (before any therapy). This may increase during the course of the disease to 10%–40% and can be even found higher at autopsy studies (40%–50%). The clinical presentations are variable according to the primary tumor site, disease stage, local and regional control, duration of follow-up, histological type, and delivered treatment. High-risk features include hypopharyngeal origin, advanced locoregional disease characterized by large tumors and extensive lymphadenopathies, poor histological differentiation, and the presence of extracapsular spread (2). Moreover, advanced age, black race, and radiological evidence of low jugular, posterior triangle, paratracheal, and contralateral lymph nodes were associated with increased risk of metastases (3, 4). The aforementioned features hold true for the most frequent histological type, i.e. squamous cell carcinoma, which will also be the principal subject of this article unless otherwise specified. Furthermore, some tumors both within this group, such as basaloid squamous cell carcinoma, and beyond, such as nasopharyngeal, adenoid cystic, and neuroendocrine carcinomas, are known for even a higher propensity to develop distant lesions (5). Typically involved sites are the lungs (70%–85% of patients with metastases), albeit a distinction from a primary pulmonary tumor can be challenging, then the bones (15%–39%) and liver (10%–30%), while skin (10%–15%) and brain (about 5%) affections remain less frequent (2). They usually occur within 2–3 years of diagnosis with the notable exception of a small proportion (probably more than 10%) of human papillomavirus (HPV)-positive oropharyngeal cancer cases, which continue to metastasize for a longer period of time, even beyond 6 years (6, 7). Interestingly, in a recent meta-analysis of seven studies, time to distant progression was 0.2–106 months and 0.2–33 months in HPV-positive and HPV-negative oropharyngeal cancer patients, respectively (8).

*How to detect them?* This is the pivotal question because imaging modalities differ in their diagnostic accuracy, which is partially responsible for the higher incidence of macrometastases found at autopsies than radiological surveys (5). In addition, our knowledge of micrometastases sometimes identified in tissue specimens remains elusive, including their clinical significance. Currently, fluorine-18-fluorodeoxyglucose (FDG) positron emission tomography with or without simultaneous computed tomography (PET/CT) scanning represents the optimal modality for detection of head and neck cancer distant spread at initial staging (9, 10). In a prospective trial of 233 patients, the addition of FDG-PET to a conventional work-up (physical examination, head and neck CT or magnetic resonance [MR], and thoracic CT) changed the M-stage in 8.6% of the study cohort (11). On the other hand, its role in follow-up of head and neck cancer survivors has been proved only for an early evaluation of regional control after definitive chemoradiotherapy but still needs to be defined for metastatic disease (12–14). One of the new, promising techniques that could find its place especially in surveillance protocols is liquid biopsy. It is based on early detection of circulating tumor deoxyribonucleic acid (DNA) mostly in the blood but also in saliva and other body fluids (15). Finally, special attention should be paid to patients with polymetastatic disease on systemic therapy, in whom close response monitoring by CT or PET/CT scans is usually performed every 6 to 12 weeks for a timely detection of disease progression, which in some highly selected cases may be treated with local ablation.

*What are the treatment options?* Although the traditional approach of oncology care in patients with metastatic tumors relies on systemic treatment with palliative intent, mounting evidence has demonstrated the utility of local ablation in certain clinical situations. Surgical resection of metastases, especially in the lungs, has been known to medical professionals since the 19^th^ century. In 1882, Weinlechner removed sarcoma metastases localized near the primary tumor infiltrating the thoracic wall. However, the first pulmonary metastasectomy as a planned and separate procedure was carried out by Divis in Prague in 1926 (16). When in 1995 Hellman and Weichselbaum thus coined the term “oligometastatic state”, surgery had already been widely accepted as a curative approach to a rather small proportion of patients, typically with lung metastases from soft tissue sarcomas, osteosarcomas, and renal cell cancers and with hepatic metastases from colorectal cancer (17). More recently, the armamentarium of local approaches has been complemented by radiotherapy and thermal ablation treatments, such as radiofrequency ablation or cryotherapy, which spare patients from a more invasive procedure at the cost of unknown treatment margins. At present, patients with oligometastatic disease of various origins are routinely offered such a potentially curative treatment, sometimes using a sequential combination of different modalities, planned in a stepwise fashion and even repeatedly in the case of accessible recurrences (18, 19).

## Approach to Oligometastatic Disease

**Figure 1:**
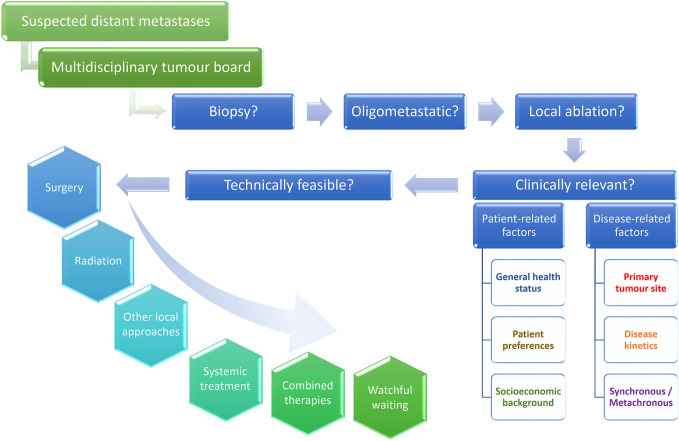
Multistep process of decision making in oligometastatic head and neck cancer patients. Patients with suspected hematogenous spread should be discussed at tumor board meetings in order to decide whether a biopsy confirmation is needed and whether local ablation can be proposed in case of an oligometastatic manifestation. Such treatment should not only be technically feasible but also clinically sound. Metastasectomy remains the treatment of choice and could be replaced by stereotactic body radiation or other local therapies in patients not suitable for a surgical intervention. Incorporating chemo- or immunotherapy or both, systemic treatment can be combined with local ablation according to the clinical setting. Watchful waiting is reserved for highly selected cases, usually as a temporary solution in heavily pre-treated patients with known disease kinetics.

Over the past year, growing efforts have been undertaken to define oligometastatic disease and its different states in order to standardize reporting thereof (20, 21). As a result, the following two conditions must be met: the maximum number of five metastases should not be surpassed, and all of them must be safely treatable, whereas a controlled primary is optional (21). According to the timing of its appearance, several distinct clinical presentations, discussed further in the text, are recognized (20–22). Since a standard approach has not been determined in these situations, all patients who could potentially be considered for a local approach should be discussed in multidisciplinary tumor boards. In this regard, we propose a multistep evaluation procedure respecting not only the technical feasibility of a given procedure but also its clinical relevance (**Figure 1**). While the former aspect is beyond the scope of this paper, we will address important pre-treatment factors here, some of which are specific for head and neck cancer, and then outline the main treatment modalities, among which the surgical approach is grounded in the strongest body of scientific evidence, followed by stereotactic radiotherapy reserved for inoperable cases. In the last section, we will deliberate over the intriguing role of combining systemic treatment with local therapies. The key message is that primary intent of these therapeutic endeavors is curative, although they may also be beneficial in consolidating response to systemic palliative treatment or postponing initiation or change thereof. Finally, the advantages of active approach should be weighed against watchful waiting, particularly in heavily pre-treated patients with repeatedly recurring and slowly progressing oligometastases.

### Clinical Pre-Treatment Considerations

The following three patient-related factors should be acknowledged before performing a planned intervention, feasible from a technical point of view.

*General health status.* The majority of head and neck cancer cases occur in the elderly, and global epidemiological projections predict increasing proportions of older people, people with cancer, and also older people with head and neck cancer, which further stimulates the strengthening position of geriatric evaluation in oncology practices. Prior to a tumor-directed treatment, all cancer patients of 70 years of age or older should undergo a frailty screening test and, according to the result, be subjected to a comprehensive geriatric assessment comprising a thorough evaluation of functional status, comorbidities, cognition, nutritional status, social support, psychological state, and polypharmacy (23). These variables are relevant, albeit to lesser extent, to the younger counterparts as well, who have usually an overall higher life expectancy and functional reserve capacity, so that the medical assessment is often limited to the appraisal of performance status and comorbidities (24). Of note, frailty, characterized by at least three of the following five criteria: weakness (grip strength), slowness, low physical activity, exhaustion, and weight loss, or pre-frailty (comprising only one or two of these criteria) can develop even in younger patients, particularly in the presence of chronic diseases, socioeconomic deprivation, and specific lifestyle behaviors (smoking, obesity) (25). Estimating overall health status of an individual and detecting unknown deficits help select an appropriate local therapy and decide on its timing and possible combinations with systemic treatment.

*Patient preference.* Shared-decision making with a well-informed patient should be encouraged whenever possible and has particular importance in borderline cases, such as when watchful waiting is proposed. Sometimes, patients can decide whether they opt for an invasive procedure or radiotherapy or thermal ablation if assumed equipotent in a given situation.

*Socioeconomic background.* It has been well recognized that socioeconomic and other disparities negatively impact on cancer incidence and survival due to associated inequalities in harmful lifestyle behaviors (smoking, alcohol intake, dietary patterns, physical inactivity), screening, and treatment (26). In head and neck cancer, lower income, high school education or less, and older age correlate with decreased overall and disease-free survival, at least in the USA (27). All these factors are particularly relevant in resource-limited countries. Moreover, the COVID-19 pandemic has disrupted oncologic care in many areas and amplified the pre-existing gap in its delivery (28).

In order to optimize the planned therapeutic intervention, the following three disease-related factors should be taken into account in patients deemed suitable according to the above-mentioned characteristics.

*Primary tumor site.* Among squamous cell carcinomas of the head and neck, HPV-positive oropharyngeal cancer represents a separate entity with distinct biological and epidemiological behavior (29). Not only is overall survival after distant failure longer in these patients, but about one third of oligometastatic cases in the lungs can be cured with either surgery or radiotherapy (7, 30). Noteworthy, compared with their HPV-negative; counterparts, patients with HPV-positive oropharyngeal cancer present more often with dissemination to more than two organs (about one third of cases) that can also involve unusual localizations such as the skeletal muscles, pericardial lymph nodes, kidney, or pancreatic tail (6–8). Therefore, careful evaluation and sometimes even multiple biopsies are warranted in these cases. Another notable exception sharing with HPV-positive oropharyngeal cancer the prominent trend of developing distant metastases is nasopharyngeal carcinoma (31). Although less evidence is available on using local ablation alone to treat metastatic lesions in nasopharyngeal carcinoma, improved outcomes have been noted if palliative systemic therapy for disseminated disease is complemented with radiotherapy of the primary lesion (32, 33).

*Disease kinetics*. Pace of the disease is one of the critical decision-making factors in patients presenting with distant spread (34). When applying local ablation to eradicate a disseminated cancer, the major concern is that the few visible macroscopic lesions represent merely the inception of an explosive manifestation. Logically, a follow-up imaging in 2–3 months gives us the desired answer but that’s actually what we try to avoid doing in the majority of cases, if not deemed suitable candidates for a wait-and-watch strategy, due to the following three reasons. First, lesions that can be treated now may progress in a couple of months in size rendering them unsuitable for the initially planned procedure. Second, new distant lesions may develop, and third, the patient’s condition may alter, either because of disease progression or underlying comorbidities, to an extent which can contraindicate further antitumor efforts. In some cases, distant metastases, particularly in the lung parenchyma, can be traced back on preceding imaging methods carried out even for other, non-oncologic reasons. In other cases, we may encounter oligoprogression which means that one or a few nodules progress during systemic palliative therapy while at the same time multiple other lesions remain under control. Subsequently, retrospective review of tumor size and other characteristics will help estimate the disease kinetics. However, in the majority of patients, the decisive factor is whether the metastases were detected at the time of initial diagnosis or whether they appeared in the course of the disease. These aspects are detailed in the following paragraph.

*Synchronous or metachronous metastases.* In the former scenario, partially owing to the insufficient information on tumor kinetics, patients usually receive systemic treatment in the first place, and if the disease is well-controlled, a local therapy is delivered at some point later. In the latter setting, corresponding to oligorecurrence or oligoprogression, a series of imaging studies is sometimes available allowing a more accurate appraisal of the disease pace and facilitating decisions about a single-modality local therapy. According to an arbitrary definition, metachronous metastases occur after 3 months from the initial diagnosis, which typically means that at least one radiological survey had been carried out. In this respect, it should be noted that in the majority of head and neck cancer patients treated with curative intent, no radiological surveillance is recommended as it had not consistently demonstrated survival benefit, although this does not perhaps hold true for some patient subgroups, such as with HPV-positive oropharyngeal cancer, in whom periodic imaging might be warranted (14).

### Surgical Treatment

Supported by the largest body of evidence, metastasectomy has been traditionally considered the gold standard in this setting. In 2015, a meta-analysis of 11 retrospective studies enrolling a total of 387 head and neck cancer patients calculated a 5-year overall survival rate at 29% after resection of metachronous pulmonary metastases (mostly single nodules). Various poor prognostic factors were reported in the included individual studies comprising the site of the primary tumor in the oral cavity, initial lymph node involvement, shorter interval from primary diagnosis to pulmonary dissemination, particularly if it occurred within 1 year, incomplete metastasectomy, and multiple pulmonary nodules (35). Literature on extrapulmonary surgery is less advanced but 5-year survival after resection of hepatic oligometastases may be in the same range (36). Additionally, the importance of new techniques should be brought to the forefront. In a retrospective cohort of different primary tumors, minimally invasive approaches, such as video-assisted thoracic surgery (VATS) and radiofrequency ablation (RFA), were associated with lower morbidity and similar local control and overall survival compared with an open resection (37). Besides that, surgical candidates are usually young patients in a good general condition, and this should also be kept in mind not only when making decisions in routine clinical practice but also when interpreting the results of available retrospective studies. Finally, the obtained full pathological specimen provides definitive diagnosis as well as additional material for immunohistochemical and molecular analyses if so needed. In this respect, differentiating a pulmonary metastasis from squamous cell lung carcinoma has been challenging and requires clinical and radiological inputs and in the case of oropharyngeal carcinoma also detection of high-risk HPV infection and not only p16 expression which can also be found in squamous cell carcinomas originating in the lungs, esophagus, and skin (38, 39).

### Radiotherapy

In patients who are unwilling or unable to undergo an invasive procedure or deemed to be at high risk of postoperative complications due to underlying comorbidities, stereotactic (ablative) body radiotherapy (SABR/SBRT) has emerged as a viable alternative to a standard surgical intervention. Derived from intracranial stereotactic radiosurgery, the methodology was introduced to clinical practice by Lax and Blomgren at the Karolinska Hospital in Sweden in September 1991 (40, 41). Based on delivering precisely targeted high doses of radiation in one or several fractions, the concept of SABR has been rapidly adopted by many institutions to treat mainly small lung cancers either primary or secondary, liver metastases, and later on also bone, lymph node, and other less frequent locations (42, 43). The increasing popularity has been mirrored by a steadily rising implementation in treatment protocols which is expected to continue in the coming years (44). Multiple single-arm studies as well as several randomized trials showed that SABR can improve disease-free and overall survival in the oligometastatic setting while maintaining good tolerance (45–51). However, covering different primary tumor types and organ sites, the available data remain heterogeneous (44). Furthermore, no randomized trial comparing a standard surgical approach with SABR has been conducted so far.

Until now, the largest *retrospective study* in head and neck cancer evaluated 82 cases of different histological types presenting either with synchronous or metachronous oligometastases (less than three in total) or multiple metastases in the lungs. Among 43 patients with oligometastatic squamous cell carcinomas, 1- and 2-year local control was 96% and 90%, respectively, and 1- and 2-year overall survival was 74% and 66%, respectively (52). Focusing solely on oligometastatic disease, another retrospective study reported 1- and 2-year overall survival of 78% and 43%, respectively, in 27 squamous head and neck carcinoma patients with up to five synchronous and metachronous metastases mostly affecting the lungs but also other organs encompassing the bones, liver, lymph nodes, and soft tissues. Local control of treated lung nodules was 74% and 52% at 1- and 2-years, respectively (53). Contrary to the former study, in which histopathological confirmation of the lung lesions was obtained in almost 90% of cases, in the latter one, biopsy was not mandatory prior to radiotherapy.

Regarding *prospective trials* on the efficacy and safety of SABR in oligometastatic disease, only a few are randomized phase II trials while most of them are single arm studies with just a small number of head and neck cancer patients (54, 55). In the largest one, Sutera et al. recruited 147 patients with up to five metachronous, biopsy-proven metastases visualized on FDG-PET/CT in at most three organs comprising the lungs (52%), lymph nodes (17%), bones (15%), and other sites. There was a large variety of primary tumors with more than half of them represented by lung cancer (22%), colorectal cancer (21%), and head and neck cancer (11%). Owing to an excess of early deaths, median overall survival of 17.6 months in 16 patients with head and neck cancer, out of which 11 had squamous cell carcinoma, was inferior to that observed in other primary tumor subgroups. However, the 42% 5-year overall survival yielded in this cohort compares favorably to outcomes yielded in surgical studies but can be biased by the small patient number (43). As of yet, the only randomized trial exploring the addition of SABR to a standard systemic palliative treatment according to primary cancer was the SABR-COMET phase II study with a 2:1 randomization in favor of the experimental arm. Oligometastatic state was defined by a maximum of five metachronous lesions with not more than three of them per organ. Biopsy was optional, and participants were not considered candidates for surgery. The three most frequently included primary tumors were breast cancer (18%), colorectal cancer (18%), and lung cancer (18%), which were not balanced between the two study arms. The number of head and neck cancer patients was not specified except for a short comment in the supplementary materials on a case of oropharyngeal cancer treated for a lung metastasis of 3 cm in diameter complicated by a large pulmonary abscess a year later. In the whole cohort of 99 patients, SABR enhanced 5-year overall survival from 18% to 42% which is very much in line with the previous study that reported this parameter at 43% for the entire study population. The benefit observed in SABR-COMET came at the cost of increased grade 2 or worse treatment-related toxicity (29% versus 9%) including grade 5 adverse events (5% versus 0%), albeit with no impact on quality of life as measured using the FACT-G scores (48, 49). These results are encouraging and imply that even poor performance and frail patients may be considered for SABR. Nevertheless, such assumption needs to be validated in further trials involving a larger proportion of oligometastatic head and neck cancer patients.

### Systemic Treatment

In the case of recurrent head and neck cancer not amenable to resection or irradiation, palliative systemic therapy can be initiated. Hematogenous metastases per se represent a sufficient criterion for this treatment, and the available registration trials did not allow consideration of local ablative methods for their management. At present, patients are usually treated with various combinations of traditional cytotoxic drugs (5-fluorouracil, platinum, taxanes) and targeted agents (cetuximab) including also immune checkpoint inhibitors (pembrolizumab, nivolumab) according to clinical factors (biological age, disease burden, pace of the disease) and programmed cell death ligand-1 (PD-L1) expression and depending also on previous treatment lines. Interested readers are referred to two of our recent publications (1, 34). Here, we would like to point out that if treated with immunotherapy in first line, patients achieve median overall survival slightly exceeding 1 year but about one third of them can still be alive at 3 years (56). Longer follow-up data are not available yet. It is also not clear whether such treatment can indeed lead to cure, and if so, then in what proportion of patients. Concerning second-line immunotherapy, only less than 10% of patients survive 3 years (57). Importantly, no studies have shown that postponing the initiation of systemic therapy has any impact on outcome, which holds true especially for indolent and slowly progressive cases, creating thus a window of opportunity for example for local ablation strategies (34, 58).

### Combination Approaches

From the above mentioned it follows that combination of local and systemic approaches might be feasible and beneficial in terms of survival parameters. Even though rigorous evidence for that is lacking, a retrospective analysis of the National Cancer Data Base provided an indirect support by identifying patients with metastatic squamous cell carcinoma of the head and neck cancer who received systemic drugs with or without locoregional therapy. With a median follow-up of 52 months, 3,269 cases were included. In propensity score-matched cohorts, 2-year overall survival was significantly enhanced in the combined treatment arm (34% versus 21%, p<0.001). Notably, the improvement pertained merely to those who underwent high-intensity locoregional therapy (oncologic resection or at least 60 Gy of radiotherapy) and was more pronounced if such intervention was delivered early in the disease course, i.e. within the first 6 months of diagnosis than later (adjusted hazard radio: 0.26 versus 0.62) (59). These outcomes suggest the importance of not only treating the locoregional disease adequately but at the same time also synchronous metastases, opening thus avenues for possible integration of their ablation in the management of otherwise locally or locoregionally advanced disease. In this respect, induction chemotherapy may be followed by definitive chemoradiation or resection of the primary tumor with subsequent local ablation of the distant lesion or lesions if they remain well-controlled throughout the treatment (60). On the other hand, different concepts pertain to metachronous presentation. Here, local ablation can be used in parallel to immuno- and/or chemotherapy either to delay a change of systemic treatment line in oligoprogressive disease or as oligo-consolidation in responding patients to eradicate a few persisting nodules (22, 61).

Another area of research relates to the radiation-induced bystander effect, also known as the abscopal effect, which is characterized by regression of nonirradiated distant lesions (62). This phenomenon is very rare but has recently been brought back to the spotlight due to a possible synergism with immune checkpoint inhibitors (63). It is therefore of interest to explore the beneficial effect of immunotherapy combined with SABR in oligometastatic disease. A phase II trial of ipilimumab, a cytotoxic T-lymphocyte antigen 4 (CTLA-4) inhibitor, and sequential or concurrent SABR to metastatic lesions of the lungs or liver demonstrated disease control in nonirradiated tumor volume of 26% in 95 patients evaluable for response with the highest rate of 42% observed after sequential SABR to one lung lesion. This trial did not focus on oligometastatic disease but possibly enrolled some of these patients. There were only four cases of squamous head and neck carcinoma, and all of them progressed (64). The absence of radiological signs of an abscopal effect in head and neck cancer was very recently corroborated in a randomized phase II trial investigating the addition of SABR to the anti-PD-1 agent nivolumab (65). However, another report described two polymetastatic head and neck cancer patients in whom the addition of SABR to PD-1/PD-L1 inhibitors induced an abscopal effect with an overall tumor regression (66). Therefore, further confirmation is clearly needed before accepting the abscopal effect might have clinical relevance.

## Clinical Practice Controversy

Despite the advantages of local ablation across different tumor types, the applicability in head and neck cancer remains to be established. Its role in the management of synchronous metastases still cannot be generalized, and radiological post-treatment follow-up in the primary disease setting in search for metachronous metastases has not been uniformly recommended in clinical practice because of its controversial impact on patient survival and the resulting low cost-effectiveness (14). However, oligometastatic disease amenable to local treatment tends to be asymptomatic due to its typical localization in the lungs, a paucity of nodules by definition, and their limited size and appears preferably late after the initial diagnosis. Such manifestation of cancer outgrowth can thus be detected only on imaging modalities, performed either as part of radiological surveillance, notwithstanding its unclear pertinence, or perhaps less frequently for other reasons.

As a result, the key issue is to define patient populations who should be exposed to a regular radiological assessment in order to be potentially able to undergo an aggressive local treatment with curative intent, acknowledging at the same time all the individuals who take these preventive measures in vain either because they will never become metastatic or will develop a distant recurrence not eligible for local treatment because of various patient- and disease-related factors. Moreover, even if a patient finally receives local ablation, it does not automatically mean cure, and in this difficult patient population, the majority of which had undergone bi- or trimodality treatment, severe late adverse events may sometimes have even more debilitating and life-threatening consequences than disease recurrence. We also need to understand that local therapy of hematogenous dissemination is rarely applied in head and neck cancer patients. Among 934 oropharyngeal cancer cases initially managed with radiotherapy with or without chemotherapy, 15% were later diagnosed with distant metastases, 4% had oligometastases (not more than five lesions confined to one organ), and disease-free survival of 1.9 to 7.7 years was seen in 10 patients (1% of the initial cohort), all of which had pulmonary oligometastases treated in 90% with local therapies. Of note, nine of these 10 cases were HPV-positive (30).

## Conclusions

Local ablation of oligometastases gives a second chance of long-term survival to patients failing primary curative treatment, especially with colorectal and renal cell carcinomas and sarcomas. In head and neck cancer, the evidence for such benefit is less clear, and this treatment is rarely delivered in clinical practice. We still need to figure out who will derive most benefit, when the right moment is to intervene, and how to optimize our diagnostic modalities for a timely identification of potential candidates. Despite this level of uncertainty and a lack of randomized trials, we advocate using this approach in selected patients after a discussion at a multidisciplinary tumor board. At the same time, we would like to stress the importance of conducting dedicated studies for squamous head and neck carcinoma patients, particularly with HPV-positive oropharyngeal cancer. A direct comparison between surgery and SABR in fit patients seems to be indispensable for further improvement as is resolving the question of implementing local ablation early in the disease course, possibly with the help of innovative approaches to disease kinetics measurements in order to exclude an early phase of an explosive distant spread.

## Author Contributions

All authors contributed to the article and approved the submitted version.

## Conflict of Interest

PS has had in the last 3 years or has advisory relationships with Merck-Serono, Servier, and BMS and received honoraria from Merck-Serono.

JV has had in the last 3 years or has consulting/advisory relationships with: Immunomedics, Innate Pharma, Merck-Serono, Merck Sharp & Dome Corp, PCI Biotech, Synthon Biopharmaceuticals, Debiopharm, Cue Biopharma, and WntResearch and received lecture fees from Merck-Serono, MSD, and BMS.

The remaining author declares that the research was conducted in the absence of any commercial or financial relationships that could be construed as a potential conflict of interest.
